# CHARACTERIZING MEDICARE ADVANTAGE APPROACHES TO PROVIDING POSTACUTE HOME HEALTH

**DOI:** 10.1093/geroni/igae098.2157

**Published:** 2024-12-31

**Authors:** Emily Gadbois, Marguerite Daus, Christine Jones, Jamie Smith, Kali Thomas

**Affiliations:** Brown University, Providence, Rhode Island, United States; Department of Veterans Affairs, Aurora, Colorado, United States; University of Colorado, Aurora, Colorado, United States; Widener University, Chester, Pennsylvania, United States; Johns Hopkins University, Baltimore, Maryland, United States

## Abstract

Medicare Advantage (MA) is a major payer of post-acute home health care, but little is known about how MA approaches home health benefit design and provision. In-depth, semi-structured interviews were conducted with 18 leaders from MA plans and care management companies and transcribed; content analysis was used to characterize underlying themes. Across interviews, participants agreed that as long as clinically appropriate, home was the preferred setting for post-acute care. Participants described the role of MA plans and care management companies in the provision of home health care, with most taking a hands-off approach to selecting the setting of post-acute care, instead deferring to patients’ choices and citing CMS regulations restricting involvement. Other organizations had more involvement in the selection of post-acute setting, with participants saying that while they don’t “steer” or “direct” setting of care, hospital discharge planners often engaged with their staff for help with difficult to place patients. In the initiation of post-acute home health, participants reported a variety of strategies for service authorization and utilization management. Plans described various approaches to care management to complement post-acute home health services, which in some cases included engaging with care management companies to deliver this aspect of care. In discussing MA approaches to post-acute home health, participants also highlighted substantial challenges facing the home health industry, particularly staffing concerns, and especially in rural areas. Insights from this work can help policymakers understand how MA plans cover home health services and coordinate with providers.

